# Graphite Oxide and Reduced Graphite Oxide Models to Reveal the Contribution of Carbon Texture and Surface Chemistry to Hydrogen Storage and Li-Ion Battery Anode Performance

**DOI:** 10.3390/nano16010019

**Published:** 2025-12-23

**Authors:** Anna Bulátkó, Lakshmi Shiva Shankar, Szilvia Klébert, Attila Farkas, Miklós Mohai, György Sáfrán, Róbert Kun, Krisztina László

**Affiliations:** 1 Department of Physical Chemistry and Materials Science, Faculty of Chemical Technology and Biotechnology, Budapest University of Technology and Economics, Műegyetem Rkp. 3., H-1111 Budapest, Hungary; bulatko.anna@vbk.bme.hu; 2 Zalaegerszeg Innovation Park, Széchenyi István University, Dr. Michelberger Pál Út 3, H-8900 Zalaegerszeg, Hungary; warrierlakshmis@gmail.com; 3 Institute of Materials and Environmental Chemistry, HUN-REN Research Centre for Natural Sciences, Magyar Tudósok Krt. 2., H-1117 Budapest, Hungary; klebert.szilvia@ttk.hu (S.K.); mohai.miklos@ttk.hu (M.M.); kun.robert@ttk.hu (R.K.); 4 Department of Organic Chemistry and Technology, Faculty of Chemical Technology and Biotechnology, Budapest University of Technology and Economics, Műegyetem Rkp. 3., H-1111 Budapest, Hungary; farkas.attila@vbk.bme.hu; 5 Institute of Technical Physics and Materials Science, HUN-REN Centre of Energy Research, Konkoly Thege M. út 29-33, H-1121 Budapest, Hungary; safran.gyorgy@ek.hun-ren.hu; 6 Sustainability Competence Centre, Széchenyi István University, Egyetem Tér 1, H-9026 Győr, Hungary

**Keywords:** graphite oxide, reduced graphite oxide, hydrogen storage, Li-ion battery

## Abstract

After being an indispensable intermediate in the oxidative exfoliation route towards graphene, graphene oxide has gained its deserved value in materials science for numerous applications, from catalysis, through energy storage and conversion, to sensor use. In this work, three graphene oxides of tuned morphology and chemistry are used as a simplified model for porous carbon materials in hydrogen storage and as a Li-ion battery anode. The BET surface areas were, respectively, 9, 13, and 535 m^2^/g, while the corresponding O/C values from the X-ray photoelectron spectroscopy were 0.51, 0.17, and 0.12. Additionally, the samples were thoroughly characterized using scanning and transmission electron imaging, powder X-ray diffraction, thermal stability, and Raman and Fourier transform infrared spectroscopic methods. Hydrogen adsorption isotherms (−196 °C) and their comparison with nitrogen uptake revealed that pore accessibility, porous confinement, and surface chemistry, i.e., both morphology and surface chemistry, contribute to efficient adsorption. In the anode application, by contrast, surface chemistry was the single most defining factor for performance.

## 1. Introduction

Due to the fascinating versatility of their textural and chemical tunability, carbon materials have been among the most widely used vectors for environmental depollution. The massively growing need for porous carbons and the expanding area of their applications have been placing porous carbon at the focus of intensive studies. The outstanding performance of nanostructured carbons is related to the synergism of pore structure and surface chemistry. The contribution of the latter can be specified by the incorporation of non-metallic heteroatoms (e.g., B, N, O, P, S), which also may generate various induced defects. Heteroatoms or surface groups may, for example, significantly influence the wetting and acid/base properties and thus play an important role in adsorption and electrochemical applications [1,2]. The most frequent heteroatom is oxygen, which occurs in the form of carboxylic, aldehyde, alcoholic or phenolic OH groups, or ether bridges [3,4].

Adsorptive gas storage [4,5,6] or carbon-based electrodes for innovative energy storage or converting devices are among the prominent emerging applications. Owing to its high energy content (142 MJ/kg) and “green” combustion end product, water (vapor), hydrogen is currently one of the most promising fuels. It can be stored either in high-pressure tanks (30–700 MPa) or in liquid form under high-energy-consuming cryogenic conditions (−252.8 °C, 0.1 MPa). Sorption of molecular H_2_ in solid-state porous materials is an attractive and less dangerous alternative owing to its fast kinetics, good cyclability, and high adsorption capacity [4,5]. In order to make adsorption storage competitive to high-pressure compressed gas or cryogenic liquid H_2_, the U.S Department of Energy (DOE) designed technical and financial targets. The present targets for on-board adsorptive hydrogen storage systems are 6.5 wt% and 50 g/L for the gravimetric and volumetric density, respectively, in the temperature range −25–100 °C [7].

The physical adsorption capacity of H_2_ is closely linked to BET surface area, pore size, pore structure, chemical properties of the adsorbent, as well as the conditions, i.e., temperature and pressure [8]. Chahine’s rule is a widely accepted relationship, which states that, in general, 500 m^2^/g equals 1 wt% hydrogen adsorption. This is equivalent to 10 μmol H_2_/m^2^ [9].

Metal–organic frameworks (MOFs) and covalent organic frameworks (COFs) are the most promising novel adsorbents to meet these targets. MOFs uniquely stand out for their ultra-high surface area, tunable pore sizes, fast uptake and release kinetics [10].

Porous carbons are the most extensively studied traditional porous materials for hydrogen storage. They possess promising hydrogen adsorption performance due to their high specific surface area and tunable pore structure [5]. Metal nanoparticle-doped activated carbons show potential for room-temperature storage, although challenged by low capacity and agglomeration. On the other hand, porous carbon composites may possess improved hydrogen uptake and release, offering new storage solutions [11].

Among carbon materials, microporous carbons are the best, as the H_2_—carbon interaction is enhanced by confinement effects. In porous carbons with uniform pore size distribution, H_2_ adsorption is the highest when the pore width is below 1 nm [12].

In pores narrower than 0.7 nm, hydrogen molecules showed hindered rotation [13]. However, for technically relevant kinetics, a wider pore size distribution is favorable. “Real” carbon adsorbents also possess heteroatoms, at least oxygen, that can significantly enhance hydrogen storage capacity through improved binding interactions. Recently, H_2_ uptake was investigated on three resorcinol-formaldehyde carbon gels at cryogenic temperature (−263–−180 °C), revealing that equilibrium adsorption characteristics depended mainly on the pore properties. The mobility of the adsorbed H_2_ molecules was influenced by both the surface chemistry and the porosity of the carbon [14]. Experimental and theoretical studies confirmed that the adsorption of H_2_ on graphenic materials is promoted by surface epoxy, carboxyl, carbonyl, and, particularly, hydroxyl groups [15,16]. The introduction of these functional groups enhances the specific surface area and increases interlayer spacing. They also result in a significant change in the location of carbon atoms, redistributing the electron structure and thus increasing the adsorption energy. Similarly, nitrogen atoms also alter the charge distribution of surrounding carbon atoms [17].

Well-tailored porous carbons with interconnected pore morphology allow the construction of high-performance electrodes in batteries and supercapacitors [18,19]. In lithium-ion batteries, porous carbon enhances the performance by increasing active sites for Li-ion storage and movement, facilitating faster electron and ion transport through a conductive network [20,21,22,23]. The porous structure also improves the mechanical stability by buffering volume changes in the electrode material [24,25,26]. Pores also create pathways for electrolyte access, enabling rapid ion adsorption/desorption and providing a host for active materials, such as sulfur in Li-sulfur batteries [27,28]. Nevertheless, the performance of porous carbon materials, not only in the earlier-mentioned functions but in any application, is a concerted outcome of their morphology and chemical properties. When the contributions of morphology and surface chemistry are to be distinguished, it is fruitful to have model materials at hand. Two-dimensional graphene and its derivatives can serve as a convenient model. Moreover, current studies emphasize that not only porosity or general heteroatom content matters. The surface chemistry, i.e., the type, amount, and distribution of functional groups and any heteroatom doping, plays a central role in governing performance. Carbonyl, carboxyl, or hydroxyl can act as active binding or adsorption sites for Li^+^ or H_2_, while epoxy may impair electronic conductivity and induce unstable interphases under electrochemical cycling [29].

Recent studies have further highlighted the critical influence of the interplay between oxygen functional groups and textural tuning in graphene or graphite oxide (GO) derived carbons on Li-ion anode performance. Tuning the amount and type of oxygen functionalities can balance reversible Li^+^ storage and electronic conductivity, while simultaneously enhancing the stability of the solid electrolyte interphase (SEI) [30]. Nitrogen doping introduces additional active sites for Li^+^ adsorption and improves electron density in the sp^2^ network, thereby increasing reversible capacity and cycling stability in rGO-based anodes [31]. Recent reports demonstrate that the combination of selective oxygen removal, heteroatom doping, and controlled textural features, such as interlayer spacing and porosity, enables superior Li^+^ diffusion kinetics and high-rate performance, establishing a clear structure–property relationship for GO-derived anodes [32].

The latest progress in Li-ion battery research shows that the field is moving toward holistic system design that brings together stable electrolytes, smarter cathode hosts, protected lithium metal anodes, and practical cell engineering. If these approaches continue to advance, Li–S batteries could deliver much higher energy density, lower cost, and better sustainability than today’s lithium-ion systems. The most promising signs come from wide-temperature electrolytes, protective anode strategies, and more realistic pouch-cell demonstrations. While the technology is not yet ready for large-scale commercialization, the direction is clear. With integrated materials and system-level solutions, Li–S batteries are steadily moving from laboratory promise toward practical, high-energy applications in the near future.

Current research is mainly focused on solving the long-known problems that stop this technology from becoming commercial. The greatest challenges are the polysulfide shuttle [33,34], the poor conductivity of sulfur, the large volume changes that happen during cycling, and the instability of the lithium metal anode [35,36]. Electrolyte research is also moving quickly. Scientists are designing new solvent–salt combinations, additives, and adjusted solvation structures to limit polysulfide dissolution and keep interfaces stable. Wide-temperature electrolytes, such as localized medium-concentration systems, have shown much better cycling stability and fewer side reactions, even under demanding conditions [37,38]. There is a clear shift toward practical cell designs. This includes building cathodes with higher sulfur loading, using lower electrolyte volumes, and testing cells in realistic formats such as pouch cells. The work on temperature-resilient binders and better cathode–electrolyte interfaces shows that the field is now aiming for real-world performance, not just lab-scale demonstrations [37,39].

It can be concluded that the morphology and the surface chemistry of the carbon material have essential and hardly separable contributions to H_2_ storage and Li^+^ battery anode performance. However, their distinctive effect is difficult to study *per se*. Various works report either the effect of systems having methodically tailored morphology **or** gradually tuned surface chemistry [29,40,41], concluding the effect on the morphological changes **or** the extent of chemical alterations.

In this current study, we report the use of three graphite oxide samples of tuned texture and surface chemistry. The selection of the samples allows for an attempt at distinction between the influence of morphology **and** surface chemistry. GO was prepared from a natural graphite with a modified version of the Hummers wet exfoliation. Controlled reduction (thermal or chemical) of GO can selectively remove unfavorable oxygen functionalities, partially restore the sp^2^-conjugated carbon network, thereby improving electron transport, while retaining stable functional sites. At the same time, heteroatom doping (e.g., nitrogen) can introduce additional active sites and modify surface polarity and electron density, thus further enhancing gas adsorption behavior or ion adsorption/intercalation [29,30,32,42]. One of the two derivatives was obtained through thermal, i.e., physical reduction, while the other was chemically treated with an environmentally friendly reducing agent, ascorbic acid. Ascorbic acid is a biodegradable and non-toxic non-hazardous reagent aligning with green chemistry principles [43]. The reduction was enhanced by using ammonia to provide a basic environment (high pH) for efficient electron transfer. During the reduction, C-N bond defect sites are also formed, improving the conductivity in energy storage applications [44]. The reduced products are labeled as tRGO and cRGO, respectively. Prior to our application studies, the samples were comprehensively characterized with multiple techniques, including thermogravimetry (TG), microscopic scanning and transmission imaging (SEM, TEM), N_2_ adsorption, powder X-ray diffraction (XRD), Raman, Fourier transform infrared (FTIR), and X-ray photoelectron spectroscopic (XPS) methods. The two reduced GOs were planned to possess significantly different textures but apparently similar chemical composition. However, owing to the relatively low temperature of the thermal treatment, GO and tRGO have comparably low surface areas. We expected that this sample set would help to distinguish the contributions of texture and chemistry, in hydrogen storage and Li-ion battery anode applications, respectively.

## 2. Materials and Methods

### 2.1. Synthesis

A graphite oxide was obtained from natural graphite (Graphite Týn, Týn nad Vltavou, Czech Republic; average nominal particle size 63 μm, purity 99.5%) by a modified Hummers method [45,46]. A mixture of KMnO_4_, H_2_SO_4_, and H_3_PO_4_ was used for the wet oxidative exfoliation. After thorough washing [47], GO cryogel rods of diameter ca. 5 mm were prepared from the aqueous suspension containing ca. 1% GO by supercritical drying. One of the reduced samples was prepared from the washed GO suspension using ascorbic acid as reducing agent (10 g suspension + 0.352 g ascorbic acid in 100 mL solution) at pH 10 set by cc NH_4_OH. After 1 h at 95 °C, the black suspension was washed with distilled water until a neutral pH was reached [48]. The chemically reduced GO (cRGO) was freeze-dried, similarly to GO. The other reduced sample (tRGO) was obtained by thermal treatment of the GO rods in a Carbolite tubular furnace. The rods were slowly (1.5 °C/min) heated to 300 °C and subsequently allowed to cool in Ar flow [49]. All chemicals if otherwise mentioned were purchased from Merck (Budapest, Hungary). The argon gas was delivered by Linde (Budapest, Hungary).

### 2.2. Characterization

Low-temperature (−196.16 °C) nitrogen adsorption measurements were performed after 24 h of degassing at 110 °C on an Autosorb-1 (Quantachrome, Boynton Beach, FL, USA) automatic volumetric instrument. The primary adsorption data were evaluated with the Anton Paar Kaomi for NOVA (version 1.01) software. The surface area (*S*_BET_) was determined using the Brunauer–Emmett–Teller (BET) model. The pore volume was estimated from the amount of vapor adsorbed at *p*/*p*_0_ = 0.95. The pore size distribution was determined using Quenched Solid Density Functional Theory (QSDFT). Powder X-ray diffraction (XRD) data were obtained using an X’Pert Pro MPD (PANalytical Bv., Almelo, The Netherlands) X-ray diffractometer with monochromatic Cu Kα radiation (1.5406 Å) at 40 keV and 30 mA. The data were analyzed using X’Pert High Score software (version 5.2) with Bragg and Scherrer equations. Raman spectra were measured using a LabRAM (Horiba Jobin Yvon, Lyon, France) instrument with a 532 nm Nd-YAG laser and analyzed with LabSpec 5 software. Scanning electron microscope (SEM) images were made on a JEOL JSM-6380LA at 10 kV accelerating voltage without coating. The transmission images were taken by a FEI Titan Themis 200 kV spherical aberration (Cs) corrected TEM 0.09 nm HRTEM and 0.16 nm STEM resolution equipped with 4 Thermofischer “Super X G1” EDS detectors. The samples were drop-dried on TEM microgrids coated with an ultrathin carbon layer. The thermal stability of the samples was analyzed by a modified thermobalance (PerkinElmer TGS-2, Shelton, CT, USA). Approximately 1.5–2 mg of samples were heated at a rate of 2 °C/min from room temperature to 250 °C, then at 20 °C/min to 900 °C in an argon atmosphere at a 140 mL/min flow rate. Fourier transform infrared (FTIR) spectra were recorded in attenuated total reflectance (ATR) mode using a Tensor 37 (Bruker Optik GmbH, Leipzig, Germany) spectrophotometer equipped with a Platinum ATR unit A225. Spectra were collected between 4000 and 400 cm^−1^ and were compared after background correction. XPS spectra were recorded with a Kratos XSAM 800 spectrometer in analyzer transmission mode with Mg Kα1.2 (1253.6 eV) excitation. The pressure in the analysis chamber was less than 10^−7^ Pa. Survey spectra were recorded in the range of 150–1300 eV in 0.5 eV steps. The C1s, O1s, and N1s photoelectron lines were measured in 0.1 eV steps with a 1 s dwell time. The spectra were referenced to the energy of the C1s line of sp^2^ graphitic carbon, which was set to a binding energy of 284.3 ± 0.1 eV. Peak resolution was performed after Shirley-type background removal using a 70:30 Gauss–Lorentz peak shape. Quantitative analysis based on integrated peak intensities was performed using XPS MultiQuant software (version 7.83) [50].

Hydrogen adsorption isotherms were obtained at −196.15 °C with an Autosorb 1C (Quantachrome) volumetric instrument using high-purity hydrogen (99.999%). For the Li-ion storage studies, the electrochemical performance was tested in a two-electrode cell using an electrochemical workstation (VMP 300, BioLogic). The working electrodes were prepared by precisely mixing the graphenic probes and the polyvinylidene fluoride (PVDF 99.9%, Solvay) binder in a mass ratio of 95:5 in a ball mill for 1 h. After adding an exact amount of N-methyl-2-pyrrolidone (NMP), the slurry was cast on a copper foil using an automatic film applicator (BYK Gardner GmbH, Geretsried, Germany). The NMP was evaporated at ambient conditions, and the film was dried overnight in a vacuum oven at 70 °C. Briefly, 12 mm diameter circular disks cut from the dried film served as anodes for half-cell tests. The CR2032 coin-type cells (Figure S1) were assembled with utmost care in an argon-filled glove box using the as-prepared coated disk and Li metal disk, respectively, as the working and counter electrode, Whatman glass fiber as the separator, and 1.0 M lithium hexafluorophosphate (LiPF_6_) in ethylene carbonate (EC) and diethyl carbonate (DEC) (EC: DEC 1:1 *v*/*v*%) as the electrolyte. The assembled cells were closed with a manual crimping machine (MTI MSK 110).

## 3. Results and Discussion

### 3.1. Characterization of the Graphenic Samples

**Figure 1:**
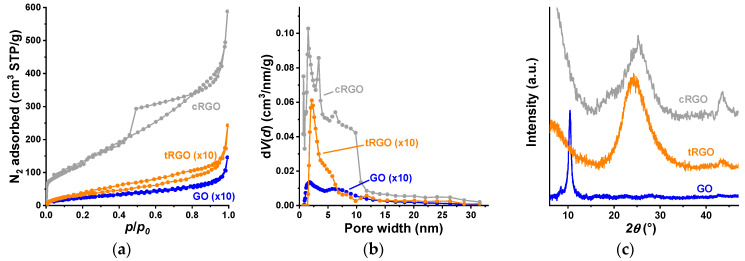
The low-temperature N_2_ adsorption isotherms (**a**), the QSDFT-based pore size distribution (**b**), and the XRD diffractogram (**c**) of the graphenic cryogels.

The slow heating rate (1.5 °C/min) and the final temperature of the thermal treatment (300 °C) were selected according to Qiu et al. [51]. The slow heating allows for avoiding the explosive thermal decomposition, i.e., it has no particular influence on the surface area. The mass of the sample hardly changes above 300 °C, implying that the reduction is practically completed. The N_2_ adsorption isotherms of GO and tRGO show a limited adsorption in the range *p*/*p*_0_ < 0.1, indicating a small amount of micropores. The predominant uptake occurs at *p*/*p*_0_ > 0.9 in the wider mesopores and macropores formed by the stacking of graphenic sheets. The nitrogen adsorption isotherms confirm that, as expected, the low-temperature thermal treatment causes no particular change in the surface area, unlike chemical reduction, which enhances the exfoliation and thus opens new surfaces accessible to the molecules of the probe gas (Figure 1a, Table 1). The shape of the isotherm in the uptake of nitrogen on an external GO surface implies a weak interaction, but in the case of tRGO, the narrow hysteresis loop, which persists also in the low relative pressure range, suggests that the desorption of N_2_ molecules is hindered. The low relative pressure range of the cRGO isotherm reveals the presence of significant microporosity. The shape of its hysteresis loop may indicate pore-blocking by narrow pore necks, while the steep upturn implies the presence of macropores with partially filled liquid nitrogen [52]. The pore volume of this sample at *p*/*p*_0_ = 0.95 is 0.66 cm^3^/g. We use the BET *C* constant to estimate the relative surface—adsorbate interaction. The α_s_ plots (Figure S2 in the Supplementary Materials) show no deviations from ideal micropore filling [52].

The strongly shifted sharp (002) graphite peak at 2θ = 10.44° in Figure 1c is typical for the strongly oxidized GO and shows that the graphite is completely oxidized [43,49]. The thermal and chemical reductions result in broader (002) diffraction peaks at 2θ = 24.37° and 25.24°, respectively, marking the efficient elimination of the oxygen-containing functional groups and, as a consequence, the partial recovery of the conjugated electron structure of graphene. The shoulder peak of the cRGO sample stems from the exfoliation of GO sheets [43]. In the reduced samples, the less intense (100) peak 2θ = 43.6° is related to turbostratic amorphous carbon components. The Scherrer formula [53] and the Bragg equation [54] were used to calculate the average height of the stacked graphenic layers *L_c_* and the interlayer distance *d*:

(1) $$L_{c}=\frac{K\cdot \lambda }{B\cdot cos\theta }$$

(2) $$d=\frac{n\cdot \lambda }{2\cdot sin\theta }$$

where *K* is a shape factor (here we use 0.9), *λ* is the excitation wavelength (nm), *B* is the full width of the peak at half maximum (FWHM) (rad), and θ is the Bragg angle. *L_c_* and *d* were used to estimate the average number *n* of graphenic layers. While in the reduced samples, the average layer distance is very close to that of the graphite, the surface oxygen function groups act as spacers in GO, leading to the divergence of the lattices.

**Figure 2:**
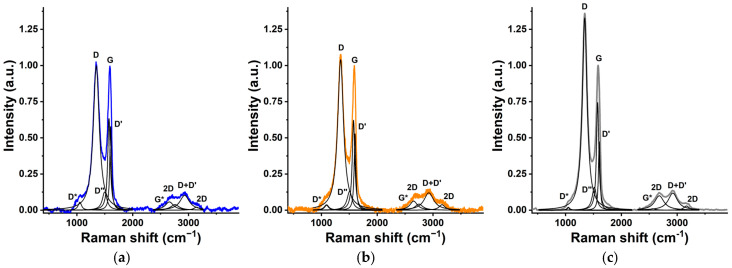
Deconvoluted Raman spectra of GO (**a**), tRGO (**b**), and cRGO (**c**).

Raman spectroscopy can also provide insight into the fine structure of graphenic systems. The spectra were recorded in the wavenumber range 200–4000 cm^−1^. The deconvoluted spectra and selected data deduced from them are shown in Figure 2 and Table 2. The most characteristic features are the iconic G and D bands, which refer to the graphitic and defect features, respectively. The G band around 1580 cm^−1^ is related to the in-plane vibration of the graphenic sp^2^ region. The D band around 1350 cm^−1^ stems from structural defects or disordered sp^2^ carbons formed, e.g., along the edges of the layers. Its intensity and position are highly sensitive to structural disorder. The position of these peaks may shift by strain or doping [55].

**Figure 3:**
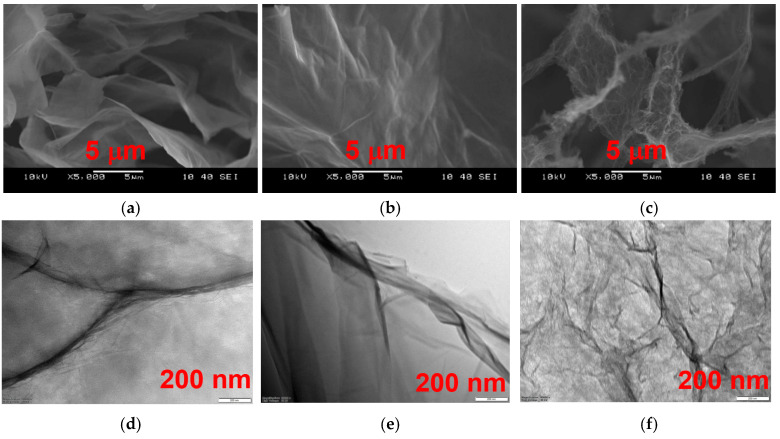
SEM images of GO (**a**), tRGO (**b**), and cRGO (**c**) aerogels. TEM images of GO (**d**), tRGO (**e**), and cRGO (**f**).

Both reduction processes increase the *I_D_*/*I_G_* ratio, indicating that the removal of the functional groups leaves behind a higher concentration of defects, particularly after the ascorbic acid treatment. The increase in the *I_D_*/*I_G_* values, particularly after the chemical treatment, is attributed to the fragmentation of the sp^2^ domains [56]. The 2D band in the overtone range, related to various phonon processes, is a marker of the quality and number of graphene layers. The ratio *I*_2_*_D_*/*I_G_*, which is strongly affected by lattice defects, corroborates again the higher concentration of defects in the chemically reduced sample [57]. The 2D bands in a dry single-layer graphene sample appear at 2679 cm^−1^. The blue shift here confirms the multilayer nature of the samples, also revealed by XRD [58]. The ratio *I_D_/I_G_* allows us to estimate the extent of the graphenic regions *L_a_* in nm as

(3) $$L_{a}=\left(2.4\cdot 10^{-10}\right)\cdot \lambda^{4}\cdot {\left(\frac{I_{D}}{I_{G}}\right)}^{-1}$$

where λ is the laser line wavelength in nm units. As expected, the reduction results in degradation of previously existing lattices and thus increases the relative contribution of defects along the edges. Morphological differences among the samples can be revealed at different length scales on the SEM and TEM images shown in Figure 3. The separated wavy multilayer GO sheets (Figure 3a) exhibit occasional wrinkles at higher magnification (Figure 3d). Both sets of micrographs corroborate the textural damage caused by the reduction. The effect of the chemical treatment is noticeably harsher, as shown by the thickened and rolled edges in SEM images (Figure 3c) as well as in the heavily wrinkled TEM micrograph (Figure 3f).

**Figure 4:**
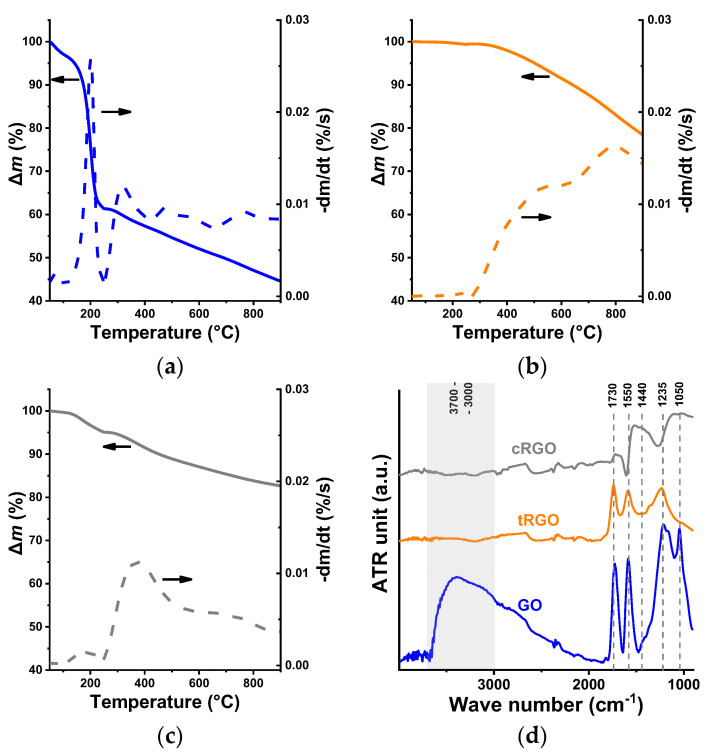
Thermal stability of GO (**a**), tRGO (**b**), cRGO (**c**), and the ATR FTIR spectra (**d**) of the samples.

The thermal stability of the three oxides reflects their chemistry and pre-history (Figure 4a–c). The stability is lowest in the oxygen-rich GO, as indicated by a sharp DTG peak around 200 °C. The thermal stability of the resulting samples was appreciably enhanced by the reductive processes, again with an easily distinguishable change after the physical and chemical treatments. The physically reduced sample is stable up to the temperature of the thermal treatment (300 °C), while the other reduced sample shows a more modest but practically continuous mass loss, with a slightly higher yield than tRGO. According to a widely accepted structural model, GO consists of intact graphenic islands dispersed with sp^3^ carbons. The sp^3^ carbons are decorated with hydroxyl and epoxide functional groups on both sides of the layers, while sp^2^ carbons may bear carboxyl and carbonyl groups, mostly along the edges of the sheets [59].

The interpretation of the Fourier transform infrared spectroscopy (FTIR) was not without challenges. This was due to the overlapping bands from numerous chemical bonds and the highly absorbent, non-reflecting nature of the material. Figure 4d shows the FTIR spectra of the graphenic samples. The assignation of the peaks is given in Table 3. All the assigned signals are much less intense in tRGO, and particularly in the cRGO samples, indicating the loss of O-containing functionalities. In accordance with the evaporation step above 100 °C in the TG/DTG signal of GO, it has a broad, intense band in the wavenumber range 3500–3200 cm^−1^ of its spectrum, corresponding to the O-H stretching vibrations. This region is completely missing from the RGOs, implying their more hydrophobic character. The decline of the C=C aromatic skeletal stretching vibrations after the reductions is aligned with the increased *I_D_*/*I_G_* ratios.

**Figure 5:**
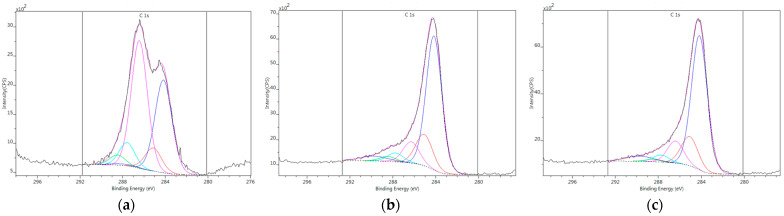
Decomposition of the C1s region of the XPS spectrum of GO (**a**), tRGO (**b**), and cRGO (**c**).

The surface composition of the samples was revealed by XPS. The survey spectra are shown in the Supplement (Figure S3). The drop in the O/C ratio after the reduction is in good agreement with the limited signal intensity in the 1050–1750 cm^−1^ region of the FTIR spectrum. The resolution of the C1s peak was performed after Shirley-type background removal using a 70:30 Gauss–Lorentz peak shape. The results of the decomposition of the C1s regions are shown in Figure 5 and Table 4 [50]. The assignation of the peaks is given in Table 5. The reductive treatments lead to a dramatic change in the shape of the C1s region. While the GO has a saddle-like shape, after the reductive treatments, the C1s regions show an asymmetric single peak. The elongated tale implies that in spite of the significant loss of oxygen, all the oxygen-containing functional groups, located either on the surface of the lattices or along their edges, are still present in the reduced samples, but in different concentrations from those in GO.

The concentration of sp^2^ carbons and the sp3 amorphous aliphatic carbons increased, while all the peaks related to O functionalities (C3, C4, and C5) decreased. The smallest reduction is observed in the C5 group, i.e., in the carboxyl or ester bonds. As indicated earlier with the FTIR spectra, the extent of reduction was more efficient via the chemical route, which reduced the O content by a factor of 1/3; thus, almost 60% of the carbon atoms became graphitic. The enhancement of the sp^2^ carbons is indicative of the restoration of the graphenic structure. This observation apparently contradicts the increased *I_D_*/*I_G_* ratio deduced from the Raman spectra (Figure 2, Table 2). This “inconsistency” is frequently observed during GO reduction [11,63,64,65,66]. The restored graphenic islands are small and disintegrated. The degradation is also reflected by the *L*_c_ and *L_a_* values in Table 1 and Table 2, respectively. The high number of edge defects has a significant contribution to the D band of the Raman spectrum.

XPS also revealed that the GO and tRGO contained traces of sulfur (1.2 at%) in the form of sulfate, a residue of the Hummers exfoliation. Below 600 °C, its elimination is not complete. In contrast, the cRGO sample contains 2.5 at% of N atoms, stemming from the NH_4_OH medium of the reduction, in states N1 (0.7 at%), N2 (1.2 at%), and N3 (0.6 at%), i.e., its majority is sp^2^ nitrogen (Figure S4). Nitrogen atoms alter the charge distribution of the surrounding carbon atoms. Due to their strong electron affinity, the adjacent carbon atoms become positively charged to counterbalance the nitrogen-induced charge delocalization. Interestingly, N-sites might inhibit H_2_ adsorption [67].

To summarize the results of the various characterization methods, we conclude that GO and tRGO possess very similar morphology that is entirely different from cRGO. As to the chemical behavior of the samples, GO is completely outstanding, but tRGO and cRGO also show minor, although essential, differences: cRGO is more reduced and contains 2.5 at% N atoms.

### 3.2. Application Tests

Carbon materials, among which include graphene and its derivatives, are one of the leading materials in sustainability-related applications [5,68]. Their outstanding performance is related to their complex, versatile, and adaptable textural and chemical properties. In graphenic materials, morphology and chemistry are considerably simpler than in porous carbons possessing sophisticated pore structure. They can therefore serve as model systems when the contributions of morphology and surface chemistry are to be distinguished. In this work, we employ the graphenic sample set for gas storage and Li-battery cathode applications.

#### 3.2.1. Hydrogen Storage

Owing to its low volumetric energy density and its highly inflammable and explosive chemical nature, the safe, efficient storage and use of H_2_ still raises several technological challenges. Adsorptive storage is one possible alternative when a proper porous storage matrix is available [57]. Recent neutron spin echo (NSE)-assisted temperature-dependent hydrogen adsorption measurements (−250–−190 °C) on polymer-based carbon gels of different textures and surface chemistry showed that the characteristics of equilibrium H_2_ adsorption depend principally on pore morphology, while the kinetics of uptake are affected both by the surface chemistry and the porosity of the carbon. It was also observed that the O-content reduces the initial adsorption energy [14].

**Figure 6:**
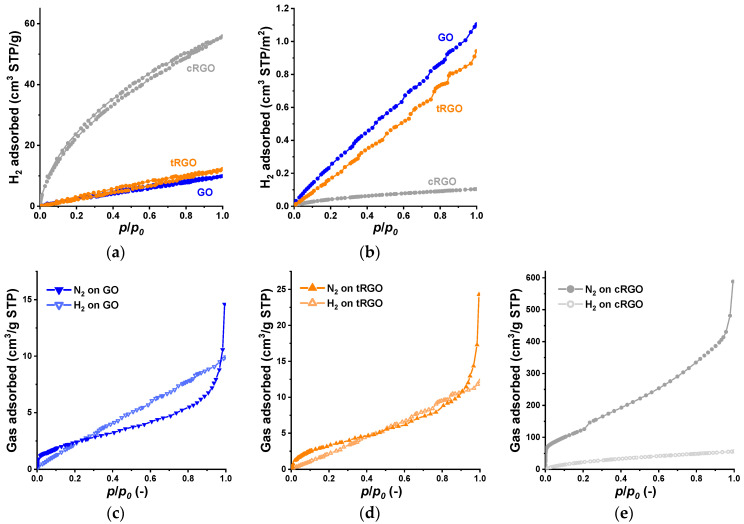
H_2_ isotherms at −196 °C of the cryogels (**a**); H_2_ uptake relative to their BET surface area (**b**); and comparison of the N_2_ and H_2_ isotherms of GO (**c**), tRGO (**d**), and cRGO (**e**).

The three graphenic cryogels were also probed for their atmospheric H_2_ capacities by measuring their H_2_ adsorption isotherm at −196 °C (Figure 6a). H_2_ uptake measured at this temperature, either at atmospheric or elevated pressure, is often used to assess the expected performance of adsorbents in hydrogen storage [11]. The measured H_2_ capacities shown here are far below the DOE target and the expected performance of potential porous materials. However, the goal of this study is not to produce prospective adsorbents but to shed light on the contribution of texture and surface chemistry to the H_2_ uptake.

The very narrow hysteresis loop that occurs over the whole relative pressure range in all three samples is a mark of hindered desorption from a confined space. The low surface area GO and tRGO cryogels display a linear, Henry-type H_2_-sorption isotherm. Only cRGO showed a concave initial section, implying a stronger attraction. The interaction-related parameter *C* from the BET fit to its hydrogen isotherm is 11 (vs. 75 for N_2_, Table 1). This sample also had the highest atmospheric uptake (55.8 cm^3^ STP/g or 5.0 mg/g), while the tRGO and GO samples gave almost identival values, 12.2 and 9.9 cm^3^ STP/g (1.0 mg/g and 0.89 mg/g), respectively. These uptakes and BET surface area (Table 1) show a similar trend.

Experimental and theoretical studies found that the adsorption of H_2_ on graphenic materials is promoted by surface epoxy, carboxyl, carbonyl, and, particularly, hydroxyl groups [15]. It has also been suggested that functional groups act as spacers and open up the interlayer void for the H_2_ molecules [69,70]. Although the O-containing functional groups and defect sites decorating GO were found to enhance the physisorption of hydrogen [68,71], the considerable difference between the chemistry of these GO and tRGO is not reflected in their volumetric H_2_ uptake (Figure 6a). In spite of the much wider *d* value of the GO (Table 1), this effect is not observed here. It is possible that the functional groups occupy part of the space between the layers, thereby reducing the interlayer volume. According to Figure 6b, the hydrogen uptake related to the BET surface area increases linearly in all three cases. The higher slope of the GO plot in this representation might be related to the surface functional groups.

As the H_2_ and N_2_ isotherms were measured at the same temperature, their respective comparison offers further information. Figure 6c–e compare the N_2_ and H_2_ uptake isotherms at −196 °C, up to atmospheric pressure. According to the pore size distribution curves in Figure 1b, the most frequent pore size accessible for N_2_ is 1.8, 2.1, and 1.5 nm in GO, tRGO, and cRGO, respectively. The kinetic diameter of hydrogen is 0.289 nm, and that of nitrogen is 0.364 nm [72]. Room-temperature in situ XRD studies revealed that at low concentration (low pressure), the H_2_ atoms attach to the external defect sites and only enter the pore at an elevated pressure [71].

In spite of the enhanced layer distance, the oxygen-rich interlayer space in GO seems to be inaccessible to N_2_. The functional groups may occasionally block the entrance and occupy part of the internal void; therefore, adsorption occurs on the external surface (Figure 6c). Although at low pressure, the H_2_ also adsorbs on the external (defect) sites, above *p*/*p*_0_~0.27, its uptake exceeds that of N_2_. This implies that the smaller H_2_ atoms have better access to the interlayer space, resulting in enhanced H_2_ adsorption, which gradually increases with pressure.

tRGO, which has a narrow interlayer space and a very similar pore size range to GO, does not display this phenomenon: the two isotherms run practically together (except at *p*/*p*_0_⟶1, due to the capillary condensation of N_2_), indicating that both gases adsorb on the external surface. cRGO shows the highest uptake of both gases, but the difference between the uptakes is 5–6 times less for hydrogen. This sample is the closest to the 1 nm pore width [12]. In spite of that, similarly to tRGO, H_2_ does not seem to have access to the interlayer space. The initial section of the cRGO isotherm suggests that the interaction with H_2_ is much weaker than with N_2_. Both probe molecules appear to be excluded from the interlayer space, but the adsorption potential of the larger nitrogen molecules attracts them to the micropores of *d* > 0.7 nm. The limited hydrogen uptake is also related in part to the measurement temperature, where the driving force of hydrogen adsorption (adsorption heat) is much weaker than the adsorption heat of nitrogen. The weak physisorption takes place—apart from defect sites—on the partially recovered graphenic regions, where the interaction with the delocalized electrons is certainly weaker than with the O-containing functional groups. Pores are far from saturation when atmospheric pressure is reached. Our findings support the crucial role of the nitrogen-related BET surface area in the assessment of H_2_ uptake [8,11,12]. Recent Random Forest machine learning studies confirmed that, in addition to pressure, the BET surface area is the dominating parameter and is linearly correlated with the excess hydrogen uptake. Oxygen content also has a positive contribution, but the effect of pore volume is quite small, and even ultramicropores become considerable only at pressures > 1 MPa [73].

#### 3.2.2. Anode in Li-Ion Battery

**Figure 7:**
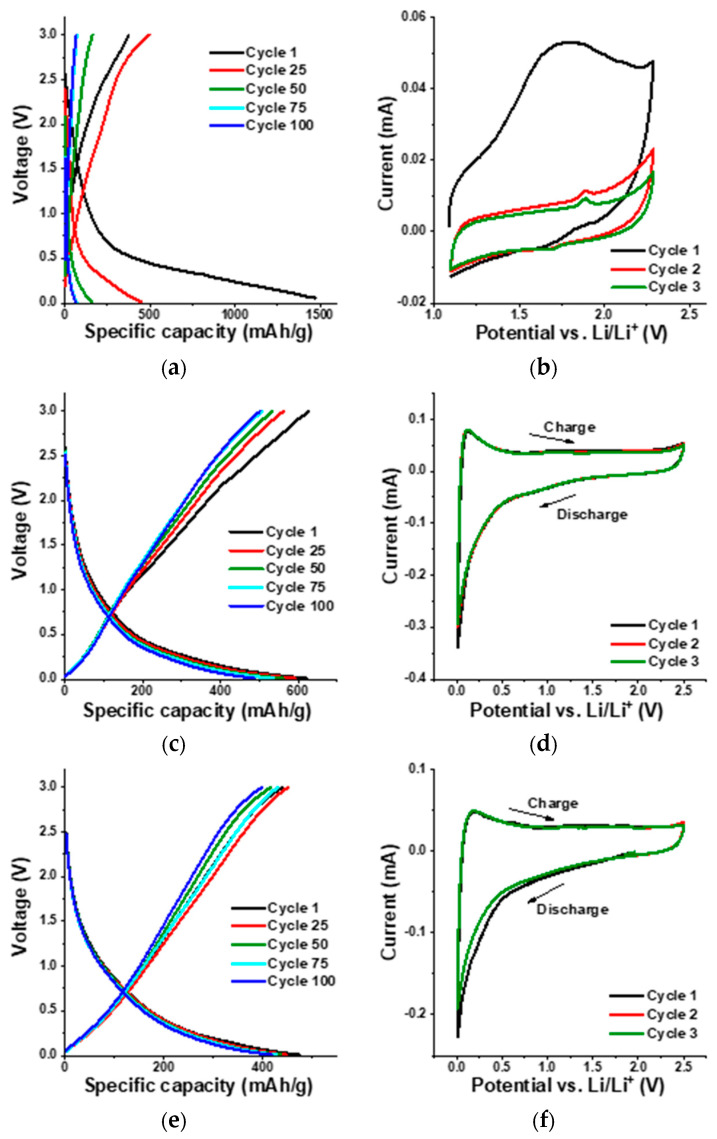
Galvanostatic charge–discharge profiles at current density 100 mA/g of GO (**a**), tRGO (**c**), and cRGO (**e**); cyclic voltammograms at scan rate 0.1 mV/s of GO (**b**), tRGO (**d**), and cRGO (**f**).

Although graphite is still the most commonly used anode in Li-ion batteries, novel nanostructured carbon materials are intensively studied as enhanced future alternatives. The electrochemical performance of the half-cells prepared from these three samples was tested in a two-electrode cell using an electrochemical workstation (VMP 300, BioLogic) at ambient temperature in a potential window of 0.01–3 V. Figure 7a,c,e show the galvanostatic charge–discharge profiles of GO, tRGO, and cRGO, respectively, at a current density of 100 mA/g. All three profiles clearly illustrate the electrochemical characteristics of graphene nanosheets, including substantial irreversible capacity loss, significant voltage hysteresis between discharge and charge, and absence of a discernible voltage plateau [74]. GO already exhibits a huge irreversible capacity loss after 25 charge–discharge cycles. The specific discharge capacity of the first cycle is not characteristic of an intercalation process; instead, it indicates a solid electrolyte interphase (SEI) formation reaction at a high voltage plateau of about 2 V. The second cycle allows for the estimation of the true specific capacity of the GO anode. In subsequent cycles, the cell goes to a low potential plateau. The voltage plateau ranging from approximately 0.8 V to 1 V is a distinct feature of graphitic anodes, where lithium typically intercalates into the layered structure of graphitic materials. The process is reversible and forms the basis for the energy storage mechanism in lithium-ion battery anodes [75,76].

**Figure 8:**
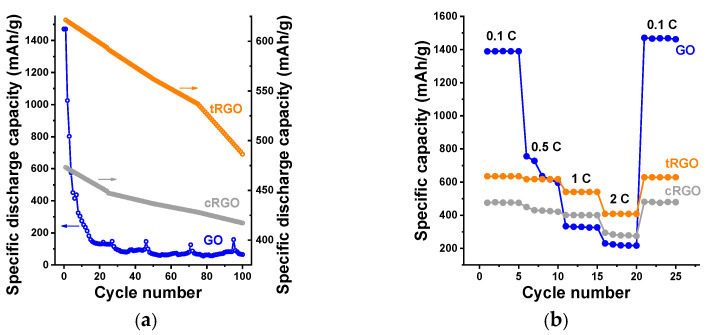
Long-term cycling performance of the three samples at 0.1 current density of 100 mA/g (**a**); rate performance of the samples at different current densities (**b**).

The cyclic voltammograms (CVs) of the GO at 0.1 mV/s scan rate are shown in Figure 7b. The peak close to 1 V is related to the intercalation of Li^+^ into the GO anode. CV curves recorded over the first three cycles indicate partial reversibility, with the first cycle showing irreversible processes associated with SEI formation and electrolyte decomposition. The CV curves (first three cycles) were recorded mainly to verify the reversibility and stabilization of the redox processes during initial cycling. The evaluation of long-term cycling stability is instead based on the galvanostatic charge–discharge tests, which were extended to 100 cycles for all samples. In agreement with the charge–discharge profile, the first cycle of the CV is different from subsequent cycles, indicating the irreversibility of the GO anode. A typical graphite anode has a specific capacity of around 372 mAh/g by forming intercalation compounds (LiC_6_). In the case of GO, even though a high initial specific capacity of 1470 mAh/g was obtained, it eventually fell to 64 mAh/g after 100 charge–discharge cycles, as shown in Figure 8a. This huge capacity loss during prolonged charge–discharge cycles can be attributed to the poor conductivity of GO, caused by its high density of oxygen-containing functional groups (hydroxyl, epoxy, carbonyl, carboxyl) that disrupt the sp^2^ network and promote irreversible reactions such as excessive SEI formation. These side reactions with the electrolyte can also lead to thick, unstable SEI layers and poor initial coulombic efficiency (~60%). In addition, GO shows a dominant capacitive contribution in the initial cycles due to the abundant oxygenated sites, but this contribution diminishes over prolonged cycling as irreversible reactions consume Li^+^ ions and block active sites [29,30]. In order to understand how the cell behaves in extreme current conditions, the rate performance of the GO anode was tested at different current densities. According to Figure 8b, the reversible capacity decreases with increasing current rate from 0.1 C to 2 C. However, the cell resumes the initial specific discharge capacity after switching the current rate back to 0.1 C.

In comparison to the GO anode, the tRGO and cRGO anodes displayed much better electrochemical properties. The initial specific discharge capacity of the tRGO anode was 621 mAh/g, which subsequently decreased to 486 mAh/g after 100 charge–discharge cycles (Figure 8a), corresponding to a capacity retention of 78%. Even though the cRGO anode exhibited a lower initial specific discharge capacity (473 mAh/g), the cell maintained a capacity of 417 mAh/g (as shown in Figure 8a), even after 100 charge–discharge cycles. This shows that cRGO anodes can deliver an 88% capacity retention, which is 10% higher than tRGO. The lower capacity retention of tRGO could be due to a kinetic limitation in the porous electrode [75]. This indicates that chemically reduced GO exhibits a much more stable charge–discharge cycling performance. Figure 8b shows the CVs of tRGO and cRGO anodes, respectively. The CV profiles of tRGO reveal stable electrochemical behavior through the first three cycles, with no significant variation, indicating excellent cycle stability. Similarly, the cRGO anode displays nearly overlapping curves between the second and third cycles, with only a slight shift observed during the initial cycle. These electrochemical responses from both tRGO and cRGO confirm their high degree of reversibility and consistent charge–discharge performance. tRGO shows improved performance compared to GO due to partial restoration of the sp^2^ network, which increases electronic conductivity and reduces polarization. Although tRGO retains some residual oxygen sites that participate in pseudocapacitive reactions, their concentration is much less than in GO. That explains the reduced irreversible capacity loss, stabilized SEI, and the initial coulombic efficiency (~98% after 10 cycles). Like the GO sample, the rate performance analysis was conducted in tRGO and cRGO anodes. According to Figure 8b, even at a high current rate, both tRGO and cRGO anodes exhibit higher reversible discharge capacities than conventional graphite anodes, indicating strong electrochemical performance.

Notably, cRGO demonstrates superior cycling stability, high-rate charge/discharge efficiency, and enhanced reversible capacity. This enhanced cycling stability could be attributed to the presence of nitrogen in cRGO. The higher electronegativity of nitrogen compared to carbon leads to a positive charge density, resulting in enhanced electrical conductivity, surface wettability, and polarity, and consequently improving the rate capability in Li-ion battery anode [77]. In addition, nitrogen incorporation promotes rapid ion diffusion, accelerates reaction kinetics, and increases the availability of Li-ion storage sites, leading to more efficient and robust electrochemical behavior.

Among the three analyzed samples, both tRGO and cRGO show potential as anode materials for Li-ion batteries. However, cRGO stands out with greatly enhanced electrochemical cycling stability. Our findings highlight cRGO as the optimum choice, providing a prolonged cycle life and exceptional anode stability, making it the most promising candidate for high-performance, long-cycle life Li-ion batteries. To enable a more direct evaluation of the electrochemical behavior of the three materials, the principal performance metrics of GO, tRGO, and cRGO are compiled in Table 6. This summary brings together the initial discharge capacities, long-term cycling outcomes, capacity retention values, and characteristic features observed in the CV profiles. The comparison clearly highlights the superior stability and reversibility of the cRGO electrode, reflecting the beneficial effects of its reduced oxygen content, more ordered graphitic domains, and nitrogen incorporation achieved during chemical reduction.

The differences in performance among GO, tRGO, and cRGO highlight the critical role of surface chemistry. In GO, abundant oxygen functional groups promote irreversible reactions and slow Li^+^ transport. Partial reduction in tRGO restores conductivity and preserves reversible Li^+^ storage sites. Nitrogen doping in cRGO further improves surface polarity, electron density, and ion mobility, leading to higher cycling stability, rate capability, and reversible capacity. In cRGO, nitrogen doping (2.5 at%) introduces pyridinic, pyrrolic, and graphitic N species that act as additional active sites for Li^+^ adsorption, enhancing the binding energy and facilitating faster intercalation kinetics. Nitrogen atoms also modify the local electronic structure of the carbon network, increasing electron density in the sp^2^ domains, which improves electronic conductivity and reduces polarization during cycling. Furthermore, N-doping can create defects and edge sites that not only serve as Li^+^ reservoirs but also improve electrolyte wetting and surface polarity, leading to enhanced rate capability and long-term cycling stability. These same factors, controlled functional groups, sp^2^ network restoration, and heteroatom incorporation, not only enhance Li-ion intercalation but also improve hydrogen adsorption by providing favorable binding sites and better pore accessibility [29,31,42,78,79,80].

In the last few years, the promise of nitrogen-doped and reduced graphene (or few-layer graphene) derivatives as high-performance anode materials for lithium-ion batteries has been reinforced. N-doped few-layer graphene maintained 493 mAh g^−1^ reversible capacity over 100 cycles at a modest rate [81]. An N-doped reduced GO demonstrated 519 mAh g^−1^ at 100 mA/g with good rate capability [82]. More recently, optimized N-doped reduced GO (with carefully tuned oxygen content and doping parameters) was proposed as a route to achieve both high capacity and improved Li^+^ ion transport, illustrating that rational design of surface chemistry and structure remains central to progress in standalone graphene anodes [83]. Reviews of graphene-based anode materials and hybrids reaffirm that while composite systems (graphene + Si, metal oxides, etc.) often outperform pure graphene, there remains clear value in understanding and improving graphene/reduced GO itself for long-cycle, high-rate performance [31]. Within this landscape, our chemically reduced, N-doped graphene (cRGO), delivering 473 mAh g^−1^ initial discharge with ~88% capacity retention over 100 cycles, stands as a competitive and realistic example of a standalone graphene-based anode. By combining sp^2^ network restoration, reduced oxygen content, and nitrogen doping, our results align with and contribute to the recent advances in the field.

## 4. Conclusions

In order to reveal the contributions of morphology and surface chemistry to the performance of porous carbon materials, graphite oxide and its two reduced derivatives were used as simplified models in hydrogen storage and Li-battery anode applications. The morphology and the surface chemistry of the three graphenic materials were carefully tuned and proven by several characterization methods, including low-temperature nitrogen adsorption, TEM and SEM imaging, XRD, Raman, FTIR, and XPS techniques. Hydrogen adsorption isotherms (−196 °C) and their comparison with nitrogen uptake revealed that pore accessibility, porous confinement, and surface chemistry, i.e., both morphology and surface chemistry, contribute to the efficient adsorption. On the other hand, in the Li-ion battery anode application, surface chemistry was the most defining factor in the performance.

## Figures and Tables

**Table 1:** Textural characteristics of the parent GO and its reduced derivatives.

Sample	*S*_BET_	*C*	Position of (002) Peak	FHMW	*L*_c_	*d*	*n*
	m^2^/g	-	°	°/rad	nm	nm	-
**GO**	9	35	10.44	0.60/0.010476	13.3	0.85	15.7
**tRGO**	13	26	24.37	5.76/0.100512	1.41	0.37	3.6
**cRGO**	535	75	25.24	5.48/0.095626	1.49	0.35	4.9

**Table 2:** Structural parameters of the cryogels from Raman spectroscopy.

Sample	Peak Position			Intensity Ratio		*L_a_*
	D	G	2D	*I_D_*/*I_G_*	*I*_2_*_D_*/*I_G_*	
	cm^−1^					nm
**GO**	1348	1576	2766	1.57	0.006	12.2
**tRGO**	1344	1576	2750	1.67	0.07	11.5
**cRGO**	1344	1575	2681	1.79	0.15	10.8

**Table 3:** Assignation of the ATR-FTIR bands [60,61,62].

Wavenumber	Assignation
cm^−1^	
**1050–1045**	νC-O-C asymmetric stretching of epoxy groups
**1235–1210**	νC–OH stretching vibrations
**1440–1430**	=C-H in-plane bending, C-O-H in-plane bending vibrations of carboxyl groups
**1585–1530**	C=C aromatic skeletal stretching vibrations of O-free graphitic domains
**1740–1730**	νC=O stretching vibration of carbonyl and carboxyl groups
**3700–3000**	νO-H stretching vibrations

**Table 4:** Distribution of the carbon species on the surface.

Sample	C	O	N	C1s					O/C	N/C
	at%			C1	C2	C3	C4	C5		
				%						
**GO**	65.6	33.1	-	30.2	8.4	50.6	7.8	3.0	0.51	-
**tRGO**	85.7	14.3	-	65.8	16.6	10.7	4.4	2.5	0.17	-
**cRGO**	86.9	10.6	2.5	68.0	15.8	11.5	3.6	1.2	0.12	0.03

**Table 5:** Assignation of the decomposed XPS peaks.

Atom	Symbol	Binding Energy	Assignation
		**eV**	
**C1s**	**C1**	284.2 ± 0.1	sp^2^ C-C
	**C2**	285.1 ± 0.05	sp3 C-C amorphous, aliphatic
	**C3**	286.4 ± 0.1	C-O-C ether, epoxy, C-OH hydroxyl
	**C4**	287.7 ± 0.1	C=O carbonyl bond
	**C5**	288.7 ± 0.2	C in carboxyl or ester bond
**N1s**	**N1**	398.4 ± 0.1	sp^2^ N in pyridine ring, C–N–C
	**N2**	399.4 ± 0.1	sp^2^ N in pyrrol or diazine ring, C≡N, N–C=O
	**N3**	400.6 ± 0.2	N in graphene plane, N–COO, O=C–N–C=O

**Table 6:** Summary of key electrochemical performance parameters.

Sample	Initial Discharge Capacity	Capacity After 100 Cycles	Capacity Retention	Key CV Features
	mAh/g	mAh/g^−1^	%	
**GO**	1470	64	4.4	Broad, ill-defined redox peaks; large irreversible capacity; poor overlap between cycles; indicates sluggish Li^+^ kinetics and unstable SEI formation.
**tRGO**	621	486	78	Better-defined peaks; noticeable improvement in reversibility from cycle 2 onwards; partial restoration of sp^2^ network visible in peak sharpening.
**cRGO**	473	417	88	Highly overlapping 2nd–3rd CV curves; sharp and stable redox features; fastest kinetics; most reversible lithiation/delithiation process among the three.

## Data Availability

The raw data supporting the conclusions of this article will be made available by the authors on request.

## Supplementary Materials

The following supporting information can be downloaded at: https://www.mdpi.com/article/10.3390/nano16010019/s1, Figure S1. Scheme of the coin cell assembly; Figure S2. a_s_ plot of the nitrogen adsorption isotherms (a) and the corresponding fits (b); Figure S3. XPS survey spectra of GO (black), tRGO (red) and cRGO (blue). At this spectral resolution, there is no significant difference between the samples (and not because of the low graphical resolution). The different background shape is due to the physical state of the sample (e.g., particle size and density) not due to its chemistry; Figure S4. Decomposition of the N 1s region of cRGO.
