# Supplementary material for: Graphite Oxide and Reduced Graphite Oxide Models to Reveal the Contribution of Carbon Texture and Surface Chemistry to Hydrogen Storage and Li-Ion Battery Anode Performance
Source: Nanomaterials (Basel). 2025 Dec 23;16(1):19. doi: 10.3390/nano16010019 (PMC12787793; doi:10.3390/nano16010019)
Supplement: Supplementary file 1 [file nanomaterials-16-00019-s001.zip › nanomaterials-4007657-supplementary.pdf]

SUPPLEMENT

Graphite oxides as models to reveal the contribution of carbon texture and surface chemistry in hydrogen storage and Li-ion battery anode performance

Anna Bulátkó[1], Lakshmi Shiva Shankar[2], Szilvia Klébert[3], Attila Farkas[4], Miklós Mohai[3], György Sáfrány[5], Róbert Kun[3,6], Krisztina László[1*]

[1]Department of Physical Chemistry and Materials Science, Faculty of Chemical Technology and Biotechnology, Budapest University of Technology and Economics, Műegyetem rkp. 3., H-1111 Budapest, Hungary.
[2]Széchenyi István University, Zalaegerszeg Innovation Park, Dr. Michelberger Pál út 3, H-8900 Zalaegerszeg, Hungary.
[3]Institute of Materials and Environmental Chemistry, HUN-REN Research Centre for Natural Sciences, Magyar tudósok krt. 2., H-1117 Budapest, Hungary.
[4]Department of Organic Chemistry and Technology, Faculty of Chemical Technology and Biotechnology, Budapest University of Technology and Economics, Műegyetem rkp. 3., H-1111 Budapest, Hungary.
[5]Research Institute for Technical Physics and Materials Science, Institute of Technical Physics and Materials Science. HUN-REN Centre of Energy Research. Konkoly Thege M. út 29-33, H-1121 Budapest, Hungary.
[6] Sustainability Competence Centre, Széchenyi István University, Egyetem tér 1, H-9026 Győr, Hungary

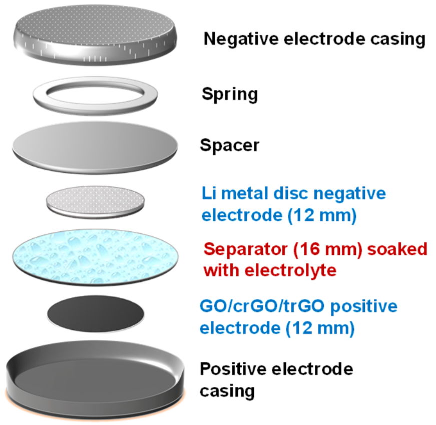

Figure S1 Scheme of the coin cell assembly

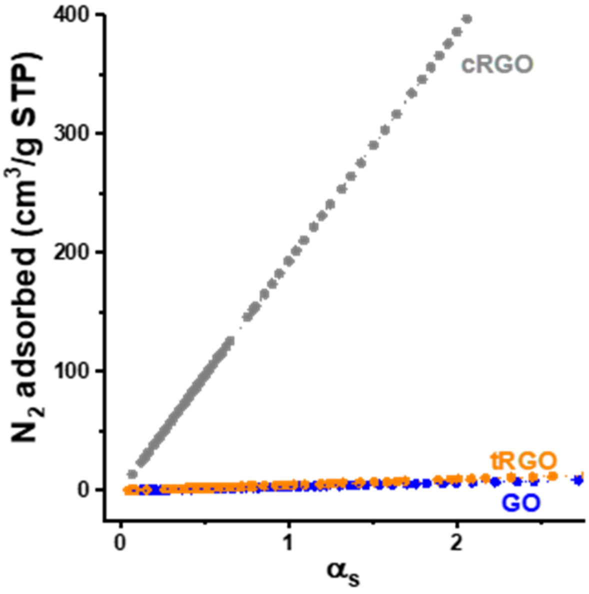

Sample	N_2		
	slope	intercept	R^2
GO	3.234	-1.776E-15	1
tRGO	4676	-8.882E-16	1
cRGO	192,996	-8.527E-14	1

a **b**

Figure S2 α_s plot of the nitrogen adsorption isotherms (a) and the corresponding fits

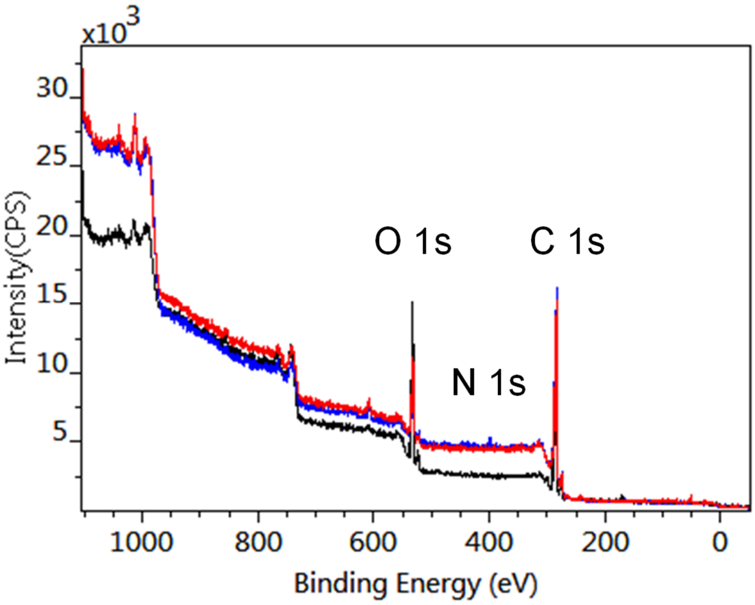

Figure S3 XPS survey spectra of GO (black), tRGO (red) and cRGO (blue). At this spectral resolution, there are no significant differences between the samples (and not because of the low graphical resolution). The different background shape is due to the physical state of the sample (e.g., particle size and density) not due to its chemistry.

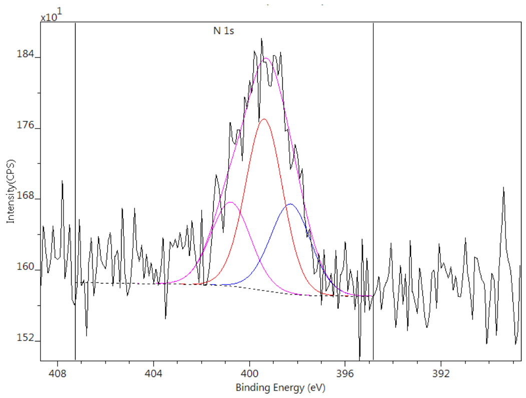

Figure S4 Decomposition of the N 1s region of cRGO
